# Adhesive Plaster, as a Valuable Constituent in the Composition of Various Apparatus Used in Surgery

**Published:** 1852-12

**Authors:** D. Gilbert

**Affiliations:** Professor of Surgery in the Medical Department of Pennsylvania College; Philada.


*Adhesive Plaster, as a valuable Constituent in the composition of various Apparatus used in Surgery.* By D. GILBERT, M. D. Professor of Surgery in the Medical Department of Pennsylvania College.

The article in the last number of the Examiner, from the pen of Professor Gross, of Louisville, Ky., on the uses of *adhesive plaster*, is valuable not only on account of the information which it embodies, but in calling the attention of the profession to the subject and eliciting its experience. The introduction of the machine-spread plaster is comparatively recent, and hence, in all probability, many valuable uses to which its convenient form has adapted it in the hands of numerous practitioners, have not yet been made public. Dr. Gross alludes to published reports of its use in the treatment of compound fractures of the bones of the leg and of fracture of the clavicle, as confirmatory of its value; and very clearly proves that the credit of first introducing the plaster, as an extending medium, is due to Dr. Swift, of Easton. Having used adhesive plaster very extensively for surgical purposes other than as a mere retentive means in wounds, I propose to furnish to the profession some of the most valuable uses to which I have applied it.

1. As Dr. Gross had evidently not read my report of the use of adhesive plaster as a *counter-extending bandage* in fractures of the thigh, as published in the *American Journal of the Medical Sciences* for January, 1851, p. 71, I infer that my account of its use may have escaped the notice of others; I would, therefore, here ask attention to what is there stated. In addition, I think it proper to state, that since then I have treated two other cases of fracture of the thigh with equal success, by means of the adhesive counter-extending bandages. In neither case was the pressure of the counter-extending bandages complained of, although the tension was kept up during the entire period of treatment. One re-application of the plaster bandages was sufficient. There was no perceptible shortening, and the undisturbed state of the fractures contributed doubtless to a more effectual and speedy union than is usually had by the ordinary treatment.

2. *Morbus Coxarius.*—In the early stages of this disease an entire investment of the parts usually covered by the splint, has been followed, in my hands, with the most pleasing results. The plaster is cut into strips of convenient length and breadth, and applied diagonally, overlapping each other, and then another layer crossing the strips of the first at salient angles. Additional strips may be applied from the pelvis to the thigh and leg, so as to restrain motion. Over this, in the cases of children, a splint made of starch and roller, fullers' boards, or any of the moulding tablets in use, may be worn to insure greater quiescence to the joint. Besides keeping the parts immoveable, the plaster acts as a mild counter-irritant, affords equable pressure, and reduces the swelling as in orchitis.

3. *Club Foot.*—In the treatment of the various degrees of varus, I use the following apparatus:

*a.* A steel sole adapted to the foot, having a process extending half an inch horizontally from the outer edge of the sole, and then rising one inch perpendicularly.

*b.* A steel leg piece, with half a circle at its upper extremity, which should be padded on the inside, by which it is attached to the leg below the knee, with buckle and strap. The process of the sole is constructed so as to receive the lower end of the leg-piece, to which it is fastened by a screw. The leg-piece has a joint opposite to the ankle, which is controlled by a screw.

*c.* A piece of adhesive plaster two and a half times the length of the foot, the width about one-fourth of the length of the plaster. Cut two-thirds of the width of this piece of plaster into strips transversely, leaving them attached to each other by the remaining third.

*Application.*—After cutting *all the tendons* at fault, at one operation, by subcutaneous section, bring the foot into its new position, and whilst held there by an assistant, apply the steel sole, and give it in charge of an assistant; then take the adhesive plaster, and apply the centre (or bight) of the uncut third to the heel, so that this is well caught, and then along each side of the foot to the dorsum, crossing over the anterior extremity of the middle metatarsal bone; carrying each extremity forward and passing it over the sides of the toes, down to the anterior extremity of the sole. The lateral strips are now drawn firmly over the sides of the foot, and made to overlap each other on the sole, fixing this firmly to the foot. The adhesive plaster and sole, thus applied, cover the foot to the extent usually occupied by a low quartered slipper. The leg-piece is next attached to the perpendicular part of the process of the sole, and buckled to the leg below the knee. Additional adhesive strips may now be applied over the foot, and extended down to the sole, so as to keep the foot firmly fixed in its new position. The foot is then elevated for a few days, and cold water may be applied, if necessary, to keep down tumefaction or inflammation. After this, the patient may be allowed to walk upon the foot. The great advantage gained by the use of this method of treatment is, that the foot is brought into its new position *at once*, and the use of the adhesive plaster obviates all the inconveniences usually experienced from pressure and abrasion when laced shoes or straps and buckles are used. The adhesive plaster becomes attached to the skin, and unites the sole, itself, and the foot, as it were into one body, and thus takes away all partial pressure and friction, and diffuses these, through the elasticity of the skin, over the entire foot and ankle. Laced gaiters made upon the steel sole may be substituted in two or three weeks.

4. *Torticollis.*—After sub-cutaneous section of the sterno-cleido muscle, in the usual manner, I use the following apparatus:

*a.* A strap of adhesive plaster one and a half or two inches wide, and long enough to reach from the temporal region of the sound side, passing close to larynx, to the temporal region of the affected side.

*b.* A portion of common suspender, tacked to the adhesive plaster, and long enough to reach from the last point named, over the vertex to the ear of the sound side.

*c.* The double tug of a suspender with its buckle.

*d.* A crescent-formed axilla pad, stuffed with any soft substance, having a button at each extremity.

*Application.*—The adhesive plaster is applied from one temporal region immediately anterior to the ear, to the other, the suspender attached is passed over the vertex, the free extremity of this is passed through the buckle of the tug, and each end of the latter is buttoned to the corresponding extremity of the axilla pad placed in the axilla of the sound side. A compress is placed over the divided ends of the muscle and confined by adhesive strips.

The pathological position of the head may now be altered at will, by drawing the free end of the suspender further through the buckle. The ear of the affected side can be made to recede from the shoulder which it had approached, and the chin to rotate from the sound shoulder, so as to correspond with and become fixed in the mesial line of the body.

*Philada.* Nov. 24th, 1852.

